# Fundamentals and applications of acrylamide based microgels and their hybrids: a review

**DOI:** 10.1039/c9ra00699k

**Published:** 2019-05-07

**Authors:** Robina Begum, Zahoor H. Farooqi, Ejaz Ahmed, Ahsan Sharif, Weitai Wu, Ahmad Irfan

**Affiliations:** Institute of Chemistry, University of the Punjab New Campus Lahore 54590 Pakistan zhfarooqi@gmail.com Zahoor.chem@pu.edu.pk; Centre for Undergraduate Studies, University of the Punjab Lahore 54590 Pakistan; State Key Laboratory for Physical Chemistry of Solid Surfaces, Collaborative Innovation Center of Chemistry for Energy Materials, The Key Laboratory for Chemical Biology of Fujian Province, Department of Chemistry, College of Chemistry and Chemical Engineering, Xiamen University Xiamen 361005 China; Research Center for Advance Materials Science, King Khalid University P. O. Box 9004 Abha 61413 Saudi Arabia; Department of Chemistry, Faculty of Science, King Khalid University P. O. Box 9004 Abha 61413 Saudi Arabia

## Abstract

Acrylamide based microgels have gained a lot of attention in the last three decades due to their potential applications in various fields based on their responsive behavior and chemical stability. In this article, the synthesis, properties, and applications of poly(*N*-isopropylacrylamide-*co*-acrylamide) [P(NIPAM-Am)] microgels and P(NIPAM-Am) microgels having an additional ionic moiety in their network [P(NIPAM-Am-IM)] are reviewed. These microgels may swell/deswell reversibly with slight changes in environmental conditions such as change in temperature/pH/ionic strength *etc.* of the medium. This responsive behavior makes the microgels a potential candidate for use in the field of nanotechnology, drug delivery, sensing and catalysis. A critical overview of the recent research progress in this area along with future perspectives is presented. The discussion is concluded with suggested possible future studies for further development in this area.

## Abstract 2

Recent advances in synthesis, characterization and applications of acrylamide based polymer microgels and their hybrids are discussed for further development in this area.

## Graphical abstract

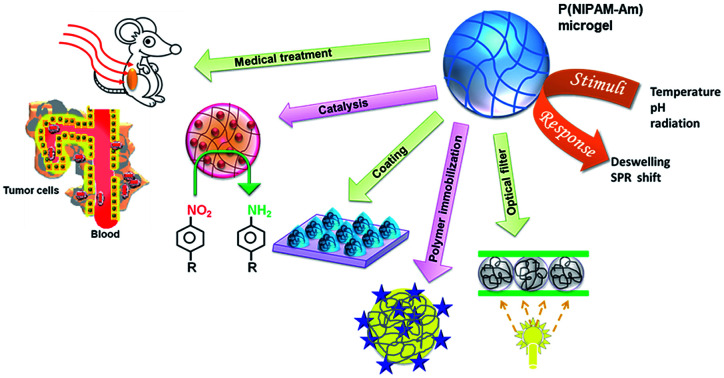

## 1. Introduction

Microgels are crosslinked polymeric particles dispersed in a suitable solvent, usually water. The diameter of microgel particles generally lies in the range of 0.1–10 μm.^1^ Microgels can be divided into various classes depending upon the nature of their feed composition. The microgels having acrylamide (Am) functionalities in their network are called acrylamide microgels. Unsaturated organic compounds of the acrylamide family are used as monomers in the synthesis of such microgels (at least one of the monomers must belong to the acrylamide family). The microgels made of Am and *N*-isopropylacrylamide (NIPAM) have been widely reported in literature.^2–6^ These microgels are temperature responsive in nature and show rapid swelling/deswelling near a certain temperature which is called the volume phase transition temperature (VPTT).^1^ Poly(*N*-isopropylacrylamide) [P(NIPAM)] is the most common temperature responsive polymer microgel system and its VPTT in aqueous medium is 32 °C.^7^ When NIPAM is copolymerized with Am, the hydrophilicity of the resultant polymeric network is increased and VPTT of the microgel system is shifted to higher temperature because Am units have more affinity towards water molecules as compared to NIPAM and it is difficult to remove water content from polymeric network made of both NIPAM and Am as compared to that made of NIPAM only.^8^ Presently, these microgels are being largely used in bio-sensing,^9,10^ drug delivery,^11,12^ catalysis^13^ and optical transduction.^14,15^ These microgels are the most promising responsive polymers that remain intact in both swollen and de-swollen states. The size of microgel particles is smaller in deswollen state as compared to that of swollen state.^16^ These microgels possess large volume for transportation/storage of various molecules. The size of mesh sieves of these microgels are in nano range, so these polymer materials can be used as templates for synthesis of metal nanoparticles.^17,18^ Metal nanoparticles can also be successfully stabilized within microgels due to donor–acceptor interaction of functionalities of polymeric system and metal nanoparticles. Size of metal nanoparticles can be controlled by controlling crosslinking density of such kind of microgel particles. NIPAM is thermo-responsive in nature which is co-polymerized with Am using *N*,*N*-methylenebisacrylamide (MBAAm) as cross linker to increase water holding capacity of the microgels.^4,19–21^ Am is a hydrophilic co-monomer^5^ and generally used in small quantity as compared to main monomer (mostly NIPAM), so it does not alter the original properties of NIPAM based polymer microgels significantly in case of its low mol percentage in feed composition of microgels but high content of Am may affect the responsive behavior of resulting microgels.^13^ Moreover, addition of Am does not increase the charge density onto the copolymer microgels to large extent due to which poly(*N*-isopropylacrylamide-acrylamide) [P(NIPAM-Am)] microgels remain non-responsive towards the pH of the medium except in very low pH values^2^ due to which these microgels do not lose their temperature sensitivity in a wide pH range. NIPAM and Am can also be polymerized with other ionic co-monomers like vinylacetic acid (VAA),^22^ phenyl boronic acid (PBA),^23^ acrylic acid (AA),^5^ methacrylic acid (MAA)^24^ to diversify their properties depending upon their application. In this review, we will mainly focus on P(NIPAM-Am) microgels. However some examples of NIPAM and Am based microgels having some additional ionic moiety in their network have been also included in this survey. The acrylamide functional group in the polymeric network is the least reactive due to which P(NIPAM-Am) microgels can be treated as inert microgel systems. The inertness of these microgel systems make them a potential candidate for the carrier of inorganic nanoparticles to be used as catalysts in various organic reactions. The addition of Am gives thermal stability to P(NIPAM-Am) microgels in aqueous medium due to its hydrophilic nature. P(NIPAM-Am) microgels have a potential to be used in catalysis due to their thermo-responsive behavior, inertness, thermal stability, open network, ligand characteristics of their functionalities, high water holding capacity, non-ionic nature and hydrophilicity in a wide temperature range and have gained a lot of interest of researchers in the last two decades.^14,23,25,26^ The polymerization of NIPAM and Am with an ionic monomer does not only increase the hydrophilicity in the network but also gives multi-responsiveness to the polymeric network.^27^ Present progress of P(NIPAM-Am)/P(NIPAM-Am-IM) microgel systems has not been reviewed critically in literature. To the best of our knowledge, properties and applications of P(NIPAM-Am)/P(NIPAM-Am-IM) microgels have not been reported in a systematic way in the form of a review article previously. Although many reviews on smart polymer microgels published by us^28^ and others^29^ are available in literature but all of those are general reviews and do not deal with this particular system of interest with respect to their properties and applications. For example Saunders *et al.* reviewed properties and biomedical applications of a wide variety of responsive microgels including P(NIPAM) microgels.^30^ It is an excellent review on use of microgel based biomaterials but it was written ten years back and is out of date now. Moreover it is a general review and covers all types of microgels. Progress in synthesis, characterization and applications of poly(*N*-isopropylacrylamide-*co*-acrylic acid) [P(NIPAM-AA)] microgels has been reviewed by our group recently^28^ but this review is related to microgels composed of NIPAM and AA monomers. Our group has been also presented development of core–shell microgels with polystyrene core and *N*-isopropylacrylamide based shell in the form of a review^31^ but microgel systems made of NIPAM and Am are not included in this report. Naseem *et al.* has published a review^32^ which emphasized on vinyl acetic acid based microgels only. Similarly a comprehensive review on responsive behavior of poly(vinylcaprolactam) has been published by Cortez-Lemus and coworkers^33^ which covers *N*-vinylcaprolactam based microgels only. Most updated, comprehensive and informative report on functional polymer microgels including few examples of acrylamide system in the form of an account has been published by Plamper *et al.*^34^ but it is also a general over view of all types of polymer microgels.

Here in, recent developments in methodologies adopted to synthesize P(NIPAM-Am) based mono-disperse microgel particles, their properties and applications have been reported. Section 1 gives a brief introduction of the topic. In Section 2, methodologies adopted to synthesize P(NIPAM-Am)/P(NIPAM-Am-IM) microgels have been discussed. In Section 3, different characterization techniques used to analyze P(NIPAM-Am)/P(NIPAM-Am-IM) microgels have been described briefly. In Section 4, temperature responsive, phase transition behavior as well as effect of acrylamide, crosslinker and surfactant feed contents on properties of these specific microgels have been discussed in detail. Applications of P(NIPAM-Am) microgels in fabrication and stabilization of inorganic nanoparticles, photonics, biomedical field and catalysis has been discussed in detail in Section 5. Summary and future aspects of this specific polymer microgel system have also been enlightened at the end of this review article in Section 6.

## 2. Synthesis of P(NIPAM-Am)/P(NIPAM-Am-IM) polymer microgels

### 2.1. Free radical precipitation polymerization

**Fig. 1:**
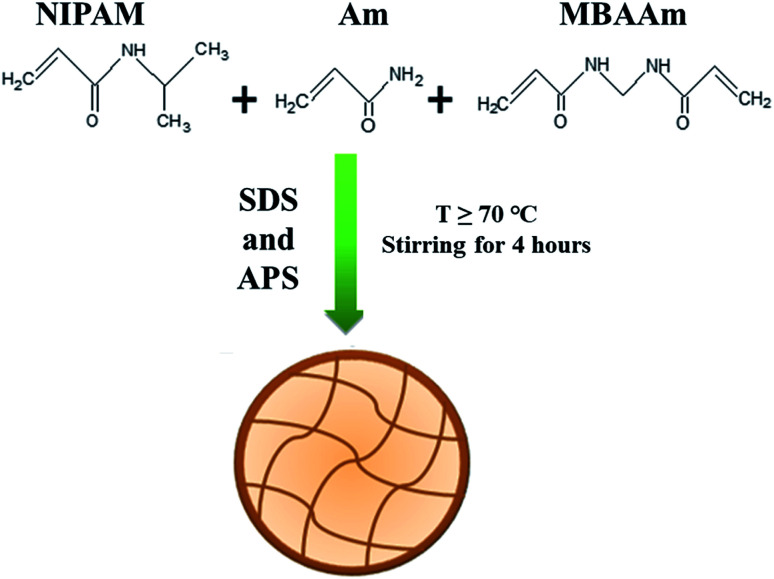
Schematic representation of synthesis of poly(*N*-isopropylacrylamide-acrylamide) [P(NiPAM-Am)] microgel by precipitation polymerization method.

The most common method used for synthesis of P(NIPAM-Am)/P(NIPAm-Am-IM) microgels is free radical precipitation polymerization.^13^ Potassium per sulfate (KPS),^12^ ammonium per sulfate (APS)^13^ or azodiisobutyronitrile (AiBN)^8^ have been reported as initiators in this method of polymerization using sodium dodecyl sulfate (SDS) as emulsifier. In this methodology, NIPAM (monomer), Am (comonomer), additional ionic monomer (if any), MBAAm (crosslinker) and SDS (surfactant) are dissolved in water. Reaction mixture is stirred to dissolve reactants in water and purged with nitrogen to remove dissolved oxygen at room temperature. Then, temperature is raised up to 70 °C and reaction mixture is further stirred at 70 °C for half an hour under continuous condensing with nitrogen supply. Then initiator generally KPS or APS is added to initiate the polymerization process. The reaction mixture turns turbid after few minutes of addition of initiator which is a sign of the onset of polymerization. Reaction is continued further for 4–6 hours under nitrogen gas purging and stirring at 70 °C.^12^ The dispersion of microgel particles is centrifuged and re-dispersed again in de-ionized water to obtain mono-disperse microgel particles. To remove surfactant, initiator and unreacted monomers, synthesized P(NIPAM-Am)/P(NIPAM-Am-IM) microgels are dialyzed against distilled water. The general scheme for synthesis of P(NIPAM-Am) microgels by precipitation polymerization is in shown in Fig. 1. Majority of the scientists have reported this method of synthesis of uniformly crosslinked P(NIPAM-Am) microgel particles with slight variation in methodology.^4,35,36^ Hoare *et al.* synthesized P(NIPAM-Am) microgels by free radical precipitation polymerization as described above and reported that this is the best method to obtain mono-disperse microgel particles.^2^ NIPAM, Am and crosslinker were successfully incorporated into the polymer microgels in stoichiometric amounts corresponding to their feed ratios. They also confirmed that NIPAM and Am were incorporated uniformly throughout P(NIPAM-Am) polymer network. It means that P(NIPAM-Am) particles of uniform composition can be obtained by precipitation polymerization of NIPAM and Am monomers using MBAAm as cross linker. They also prepared P(NIPAM-Am-MAA) microgels using the same methodology but distribution of methacrylic acid units (ionic co-monomer) was not found to be uniform throughout the polymer network by precipitation polymerization.^2^ This indicates that use of P(NIPAM-Am) microgel system is the best choice as compared to P(NIPAM-Am-MAA) microgel system where uniformity of monomer units in the network matters.

Zhang *et al.* synthesized a series of monodisperse P(NIPAM-Am) microgels using different feed contents of Am in the presence of different amounts of SDS and reported that morphology and responsive behavior of the microgels can be easily tuned using free radical precipitation polymerization method of preparation of microgels.^37^ The similar observations have been also reported by Shen and coworkers.^19^ Synthesis of monodisperse P(NIPAM-Am-IM) microgel particles with different IM units like P(NIPAM-Am-AA),^38^ P(NIPAM-Am-MAA)^39^ and P(NIPAM-Am-VAA)^17^ by precipitation polymerization method described above has been reported in literature.

### 2.2. Redox polymerization

The free radical precipitation polymerization of NIPAM and Am is generally carried out at 70–80 °C in aqueous medium as described above but polymerization can be achieved successfully at low temperature by using some accelerator. The initiator and accelerator form a redox pair to initiate radical polymerization at low temperature and this method is known as redox polymerization. Synthesis of P(NIPAM-Am) hydrogels at 4 °C using sodium persulfate (NaPS) and *N*,*N*,*N*ʹ,*N*′-tetramethylethylene-diamine (TEMED) as redox pair in aqueous medium has been reported in literature.^40^

### 2.3. Synthesis of microgel particles from P(NIPAM-Am) linear chain polymer

Synthesis of biocompatible and biodegradable microgels having P(NIPAM-Am) polymer chain as a minor component have been also reported by Sung and coworkers using totally different methodology.^11^ In this method, P(NIPAM-Am) linear chain polymer was synthesized initially. Then polymer aqueous solution was mixed with gelatin sol phase solution and emulsified with silicone oil in the presence of a triblock copolymer surfactant [poly(ethylene glycol)-*block*-poly(propylene glycol)-*block*-poly(ethylene glycol)]. Silicone oil was removed by repeated centrifugation of the mixture. After that, phosphate buffer solution containing a natural cross linker genipin was added into gelatin loaded with P(NIPAM-Am). The gelatin based thermo-responsive microgels cross linked by genipin were used for controlled drug release. A similar method of synthesis of crosslinked microspheres from pre-formed linear thermo-responsive poly(*N*-isopropylacrylamide-*co*-acrylamide-*co*-hydroxyethyl acrylate) [P(NIPAM-*co*-Am-*co*-HEA)] system has been described by Fundueanu and coworkers.^16^ They determined composition of pre-formed P(NIPAM-*co*-Am-*co*-HEA) linear polymer using ^13^C NMR spectroscopy. They used glutaraldehyde as crosslinker to convert linear polymers into microspheres.

### 2.4. Radiation polymerization

P(NIPAM-Am) hydrogels are also prepared by radiation polymerization at room temperature.^41^ Yong *et al.* prepared P(NIPAM-Am) copolymer hydrogels with different feed contents of NIPAM and Am in aqueous medium by using method of radiation polymerization.^41^ Co-60 gamma rays were used as source of irradiation. This method is used for synthesis of bulk hydrogels which grinded into small particles. Then these particles are dispersed in water for various applications. Moreover polymer particles made by this method are not monodisperse due to which their applications are limited.

## 3. Characterization of P(NIPAM-Am) based polymer microgels

Various techniques like Photo correlation spectroscopy,^12^ Transmission electron microscopy (TEM),^8^ Dynamic light scattering (DLS),^1^ UV-visible spectroscopy (UV-vis),^13^ Fourier transform infrared spectroscopy (FTIR),^42^ Scanning electron microscopy (SEM),^43^ Raman spectroscopy (RS),^8^ Energy dispersive X-ray spectroscopy (EDX),^44^ Nuclear magnetic resonance spectroscopy (NMR),^45^ Differential mechanical analysis (DMA),^46^ Laser light scattering spectrometry (LLS),^9^ Differential scanning calorimetry (DSC)^13^ and Atomic force microscopy (AFM) have been reported to characterize P(NIPAM-Am) microgel particles. Photon correlation spectroscopy/DLS is used for determination of hydrodynamic size and size distribution of polymer microgel particles. This technique is also used to investigate temperature and pH responsive behavior of P(NIPAM-Am) microgel system. Microscopic techniques like TEM, SEM and AFM are utilized for determination of morphologies of P(NIPAM-Am) microgel particles and their hybrids.^47^ NMR, FTIR and RS are widely used methods for identification of functionalities of the resulting polymer particles.^48^ EDX and XRD methods are applied to inorganic nanoparticles loaded acrylamide based microgels to confirm metallic nature of inorganic materials loaded in polymer microgels.^49^ UV visible spectroscopy is used to determine VPTT of the P(NIPAM-Am) microgels and their hybrids. Formation of Plasmonic nanoparticles inside the P(NIPAM-Am) microgel dispersion can be confirmed by UV visible spectroscopy. This technique is also useful for investigation of tuning of optical properties of P(NIPAM-Am) microgels loaded with Plasmonic nanoparticles. The progress of reaction catalyzed by metal nanoparticles loaded P(NIPAM-Am) microgels can be monitored by UV visible spectrophotometry.^13^ The potential of this technique for characterization of smart polymer microgels and their hybrids for different applications has been recently reviewed by our group.^50^ TGA, DSC and DMA are used for investigation of thermal stability of P(NIPAM-Am) microgels and their hybrids. TGA is also useful for determination of metal content in P(NIPAM-Am) microgels loaded with metal nanoparticles.^13,51^ The purpose of use of aforementioned characterization techniques has been described in detail in later sections.

## 4. Properties of acrylamide based microgels

### 4.1. Temperature responsive behavior of acrylamide based microgels

**Fig. 2:**
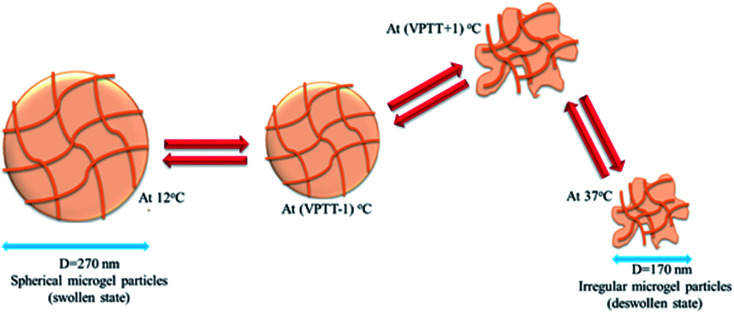
The variation in size and shape of P(NIPAM-Am) microgel particles with change in temperature.^1^

Microgel particles which show a sudden change in their size (measured in term of hydrodynamic radius or diameter) with the change in temperature of the medium are called thermo-responsive microgels and this behavior of microgels is called their thermo-sensitivity. P(NIPAM) microgels are well known temperature responsive microgels which shows sudden decrease in their size at 32 °C in aqueous medium and their thermo-responsive behavior has been widely reported in scientific literature.^52–56^ P(NIPAM-Am) microgels are also thermo-responsive due to presence of NIPAM content in their network. However Am is hydrophilic monomer and its polymerization with NIPAM may shift VPTT of resulting microgels to higher temperature in aqueous medium. Wang *et al.* measured hydrodynamic diameter of P(NIPAM-Am) microgels in the temperature range of 20–50 °C using DLS in aqueous medium and reported that the average hydrodynamic diameter of P(NIPAM-Am) microgels was decreased from 270 to 170 nm with the increase in temperature of the medium from 12 to 37 °C along with shift in VPTT depending upon feed content of microgels.^1^ Effect of feed content on thermo-sensitivity of P(NIPAM-Am) microgels has been discussed in later sections (4.3–4.6). They also investigated the size and shape variation of microgel particles with increase in temperature from 12 to 37 °C using TEM analysis and found that morphology of microgel particles was changed from spherite to irregular shape along with decrease in their size as shown in Fig. 2. The aqueous dispersion of P(NIPAm-Am) microgels was placed on carbon coated copper grid and was allowed to dry for 24 hours at aforementioned values of temperature before TEM measurement. The irregularity in shape of microgel particles at elevated temperature may be due to heterogeneity in structure of polymer network while swelling of microgel particles at temperature < VPTT is due to formation of hydrogen bonding between water and amide groups of polymer network. At temperature > LCST, hydrogen bonding between water and microgel contents was disrupted and hydrophobic interaction among the microgel contents was become stronger and as a result microgel particles were deswelled. The similar observations of variation in size of P(NIPAM-Am) microgel particles with change in temperature has been reported by other researchers.^5,57,58^ The polymerization of NIPAM, Am with an additional ionic monomer does not only increase the hydrophilicity in resulting microgels but also gives them multi-responsive behavior. Farooqi *et al.* investigated the variation in size of P(NIPAM-Am-AA) and P(NIPAM-Am-PBA) microgels with change in temperature, pH and ionic strength of medium and noted that both microgel systems shows variation in their size with aforementioned stimuli.^10^ They also observed that P(NIPAM-Am-AA) are thermo-responsive under low pH values and lose their temperature sensitivity at high pH values because of increase in hydrophilicity in polymer network as a result of ionization of carboxylic acid groups.

### 4.2. pH responsive behavior of acrylamide based microgels

The P(NIPAM-Am) microgel particles are considered irresponsive to change in a wide range of pH of the medium. For example Wu *et al.* measured hydrodynamic radius of P(NIPAM-Am) microgel particles as a function of pH ranging from 2.5 to 8.5 in aqueous medium at 22 °C using dynamic light scattering measurement at scattering angle of 90 °C and found that value of *R*_h_ did not change with change in pH of the medium in aforementioned pH range.^59^ However under very low pH (in highly acidic medium), these microgel particles may get swell due to electrostatic repulsion among positively charged amide groups of polymeric network. But highly pH responsive Am based system can be obtained by copolymerization of NIPAM and Am with any ionic monomer. For example Wu and coworkers also studied pH responsive behavior of P(NIPAM-Am-AA) microgels in the same pH range under similar conditions and found that the value of *R*_h_ increases with increase of pH of the medium and significant change was noted in the pH range of 3.5–4.5 (p*K*_a_ of AA is 4.2).^59^ Similarly Khan *et al.* reported pH responsive behavior of P(NIPAM-Am-VAA) microgels in aqueous medium.^17^ They found that the value of *R*_h_ of P(NIPAM-Am-VAA) microgel particles increases with increase in pH of the medium. The significant change in *R*_h_ of P(NIPAM-Am-VAA) microgel particles was observed in pH range of 4–5 which is very closed to p*K*_a_ value of VAA (p*K*_a_ of VAA is 4.5). This increase in radius of particles may be attributed to electrostatic repulsion among charged moieties caused by protonation of carboxylic acid groups of VAA units of polymeric network. They reported that the value of *R*_h_ becomes constant at pH ≥5 because no carboxylic acid is available for ionization and system becomes fully extended. The similar pH responsive trend of P(NIPAM-Am-VAA),^17^ P(NIPAM-Am-MAA)^39^ and P(NIPAM-Am-AA)^59^ microgels have been also reported in literature.

### 4.3. Effect of acrylamide feed content on properties of P(NIPAM-Am) microgels

**Fig. 3:**
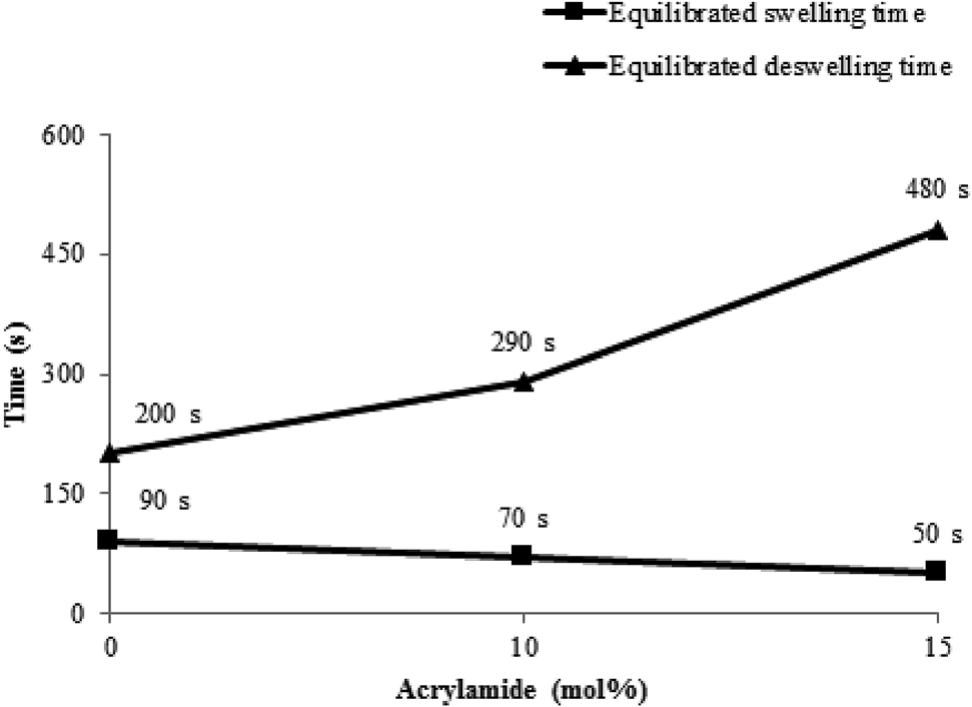
Dependence of swelling and deswelling time on acrylamide mol percentage.^1^

The feed contents of P(NIPAM-Am) microgels have effect on responsive properties of resulting microgels. This effect is generally described in the form of deswelling ratio (*α*) which can be defined as1
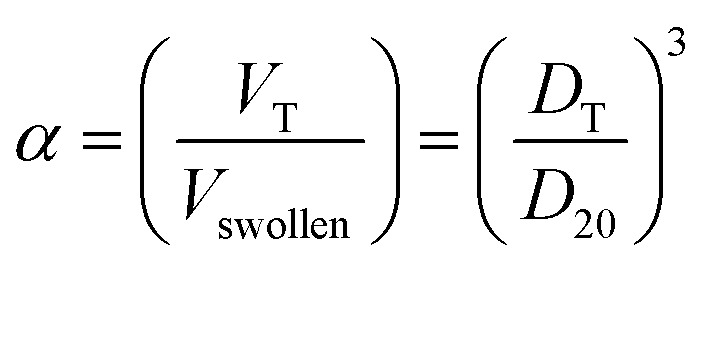
where *V*_T_ and *V*_swollen_ are the values of average volume of microgel particle at any temperature and in swollen state (at 20 °C) respectively. *D*_T_ and *D*_20_ are values of average hydrodynamic diameter at any temperature and at 20 °C (in swollen state) respectively. Values of equilibrium deswelling ratio of P(NIPAM-Am) microgel samples Am-0, Am-10 and Am-15 with 0, 10 and 15 mol percent of Am at different temperatures of the medium reported by Wang *et al.*^1^ are listed in Table 1. The time required for swelling of P(NIPAM-Am) microgels is increased with increase in AAm feed content and time required for deswelling of P(NIPAM-Am) microgels is decreased with increase in AAm feed content (Fig. 3). When Am feed content of P(NIPAM-Am) microgels was increased, VPTT of the microgel system was shifted to high value of temperature. The value of LCST/VPTT of Am-0, Am-10 and Am-15 was found to be 32, 34 and 38 °C respectively in aqueous medium. The increase in value of LCST with increase in Am content in P(NIPAM-Am) microgels has been also reported by Fang *et al.*^60^ who prepared P(NIPAM-Am) microgels with NIPAM : Am mol ratio of 83 : 17 for which value of LCST measured by UV-visible spectroscopy was found to be 40.1 °C. Budhlall *et al.* adjusted feed ratios of NIPAM and Am to obtain P(NIPAM-Am) microgels with VPTT temperature near to physiological temperature for designing of drug delivery system.^4^ Shen *et al.* also reported that P(NIPAM-Am) microgels with NIPAM : Am mol ratio of 9 : 1 has VPTT at 35 °C with a broaden transition range.^19^ Similar tuning of VPTT of P(NIPAM-Am) microgels has been also reported by Zhang *et al.*^37^ and Sanz *et al.*^61^ Thermo-sensitivity of microgel was decreased with increase of Am feed contents. The value of equilibrium deswelling ratio of P(NIPAM-Am) polymer microgels measured by Wang *et al.*^1^ was decreased with increase of temperature of the medium as shown in Table 1.

**Table 1:** Values of equilibrium deswelling ratio of P(NIPAM-Am) microgels with different mol percentages of NIPAM and Am at different values of temperature of the medium^1^

Temperature (°C)	Equilibrium deswelling ratio (*α*)		
	Am-0	Am-10	Am-15
27.5	0.90	0.98	0.99
30.0	0.83	0.91	0.93
32.5	0.72	0.84	0.91
37.2	0.39	0.45	0.75
40.0	0.39	0.39	0.53

### 4.4. Effect of crosslinker feed content on properties of P(NIPAM-Am) based microgels

Thermo-responsive properties of P(NIPAM-Am) microgels can also be tuned by changing the crosslinker feed content. Wang *et al.* also studied the effect of crosslinker feed content on thermo-responsive behavior of P(NIPAM-Am) microgel particles.^62^ P(NIPAM-Am) microgels with 0.5 to 5.0 mol percent of crosslinker feed content showed abrupt change in hydrodynamic diameter in temperature range of 35–40 °C while microgels with 7.3 mol percent crosslinker do not show such type of behavior. Thus P(NIPAM-Am) microgels with low crosslinker feed content are flexible and more sensitive to temperature as compared to microgels with high crosslinker contents. It was also observed that size of microgel particles was decreased with increase of crosslinker feed contents.

### 4.5. Effect of surfactant feed content on properties of P(NIPAM-Am) microgels

The amount of surfactant used during synthesis also affects the properties of P(NIPAM-Am) microgels. Shen *et al.*^19^ fabricated P(NIPAM-Am) microgels with NIPAM : Am mol ratio of 9 : 1 in the presence of different amounts of SDS and noted that *R*_h_ decreases significantly with increase in feed concentration of SDS at constant feed contents of NIPAM and Am. They measured *R*_h_ of P(NIPAM-Am) microgels in aqueous medium at their VPTT (37 °C) using DLS as a function of mol percentage of SDS and noticed the decrease in *R*_h_ value with increase in concentration of SDS. Size regulation of P(NIPAM-Am) microgels by SDS has been also reported by various groups.^37,63^ Effect of nature and alkyl chain length of anionic surfactant on properties of *N*-isopropylacrylamide, *N*-isopropylmethacrylamide and *N-n*-propylacrylamide based polymer microgels is beyond the scope of this review and can be seen in literature.^64^

### 4.6. Effect of ionic strength on properties of P(NIPAM-Am) microgels

It is very important to investigate the effect of ionic strength on properties of P(NIPAM-Am) microgels for their potential applications in biomedical field but unfortunately this aspect has not been described in literature in detail. Only a brief report on effect of ionic strength on the value of VPTT of P(NIPAM-Am) microgels is available in literature.^37^ Zhang and coworkers measured value of LCST/VPTT of P(NIPAM-Am) microgels in the presence of aqueous solution of different ionic strength using UV-visible spectroscopy and reported that value of LCST is decreased with increase in ionic strength due to well-known salting out effect. The value of LCST of P(NIPAM-Am) microgels with Am 15 wt% of the NIPAM was found to be decreased from 44 °C to 40 °C with increase of ionic strength from 0.0 to 0.16 M in aqueous medium.^37^ It is needed to further explore the effect of ionic strength and nature of ions and solvent systems on responsive behavior and stability of P(NIPAM-Am) microgel particles in future studies.

### 4.7. Phase transition behavior of P(NIPAM-Am) microgels

P(NIPAM-Am) microgels show phase transition with change of temperature of the medium, content of acrylamide and crosslinker in the microgels. P(NIPAM-Am) microgel particles with 2.5 mol percent crosslinker showed four transition phases while microgel with 7.3 mol percent crosslinker showed three transition phases due to loss of flexibility and decrease of swelling ratio. Wang *et al.* observed these transitions of microgels phase induced by changing temperature.^12^ The microgel system was found to be a semi-translucent swollen gel at temperature below 22 °C. Microgel was changed into clear, flowable suspension with change of temperature in range of 22–36 °C. The temperature at which clear, flowable microgel phase and swollen microgels suspension phase co-exist is called gelling temperature (GT). Flowable characteristic of microgel was in fact due to weakening of hydrogen bonding present between water molecules and microgel functionalities in result of increase in temperature. Further increase of temperature in range of 36–40 °C increased the strength of polymer–polymer hydrogen bonding as compared to polymer–water hydrogen bonding. Also, hydrophobic interactions were established between polymer chains and clear suspension turned into milky appearance. The temperature at which this phase transition was occurred is called cloud point temperature (CPT). Above 40 °C, microgel suspension was turned into shrunken state rapidly. At this stage, water come out of microgels network. This temperature is called VPTT. Further increase in temperature may cause aggregation of microgel particles. Crosslinker content of microgels also tune their phase transition behavior.

### 4.8. Phase transition kinetics of P(NIPAM-Am) based microgels

Phase transition kinetics of P(NIPAM-Am) microgels was studied by Wang *et al.* using UV-visible spectroscopy^1^ which is an excellent tool for this purpose.^50^ The value of transmittance of the microgel samples containing different Am contents measured at their VPTT was found to be decreased with time and became constant at deswelling equilibrium. The time required to attain deswelling equilibrium was increased with increase of Am contents. As Am is hydrophilic in nature and its high contents inside the polymer network interacted with large amount of water through hydrogen bonding. Thus, more time is required to expel out water from microgel to attain deswelling equilibrium. Similarly swelling kinetics of the microgel samples was studied by measuring value of transmittance at 12 °C as a function of time. The value of time required to attain swelling equilibrium was found to be decreased with increase of Am contents in the microgels. They reported that concentration of P(NIPAM-Am) microgel particles also affects the swelling/deswelling kinetics of dispersion. Deswelling equilibrium of microgel dispersion with its high concentration was attained in small time (240 s) as compared to that for microgel dispersion with low concentration (300 s). While swelling equilibrium was attained more rapidly in microgel dispersion with low concentration as compared to microgel dispersion with high concentration.

## 5. Applications of P(NIPAM-Am) based microgels

### 5.1. P(NIPAM-Am) based microgels for stabilization of inorganic nanoparticles

Polymer microgels containing NIPAM and Am have gained much attention as micro-reactors for *in situ* fabrication of inorganic nanoparticles in the last two decades due to potential applications of resulting hybrid microgels.^5,9,10,23,26,36,65^ Deep literature review reveals that NIPAM and Am based microgels containing some additional ionic moiety are widely reported for this purpose^5,66^ but synthesis of metal nanoparticles within P(NIPAM-Am) microgels has been rarely reported in literature. We are the first who reported fabrication and stabilization of silver nanoparticles in this particular microgel system using *in situ* reduction of silver nitrate (AgNO_3_) by NaBH_4_.^5^ There is no straight forward report on fabrication of metal nanoparticles within P(NIPAM-Am) microgel dispersion before our work. However polymerization/crosslinking of NIPAM and Am around a single gold nanoparticle using *N*,*N*-methylene bis acrylamide as crosslinker, APS as initiator and SDS as emulsifier has been reported.^67^ In this method, Au nanoparticles were synthesized using citrate reduction of chloroauric acid. The surface of Au nanoparticles was modified by growing a monolayer of Am around Au nanoparticles. Then NIPAM and Am were polymerized around Au nanoparticles by free radical precipitation polymerization as described in Section 2 to obtain Au@IPN-P(NIPAM-Am) core shell hybrid microgels with Au single nanoparticle core and P(NIPAM-Am) shell. The central core made of Au nanoparticles is surrounded by polymeric shell and cannot be accessed easily due to which its applications become limited. Another alternate approach of fabrication of hybrid microgel system is stabilization of metal nanoparticles on the surface of microgel particle. These particles are easily accessible due to their presence on the surface of polymer microgel particle. Fang *et al.* synthesized Fe_3_O_4_@P(NIPAM-Am) core shell composite particles with ferric oxide (Fe_3_O_4_) nanoparticle core and P(NIPAM-Am) shell and attached gold nanoparticles to the surface of Fe_3_O_4_@P(NIPAM-Am) core shell composite particles to obtain magnetic hybrid microgels.^8^ But metal nanoparticles present on the surface of polymer microgel particles are not stable enough for their potential applications. So the best strategy to obtain stable and applied hybrid microgels is fabrication of metal nanoparticles within microgels. The metal nanoparticles cannot only be fabricated in microgels but also can be stabilized for a long time due to some sort of interaction between them and functional groups of the polymeric network. P(NIPAM-Am) microgels may be a potential candidate for stabilization of metal nanoparticles due to interaction between amide groups and metal nanoparticles but stabilization of metal nanoparticles in these microgels has not been widely reported in this particular system as compared other microgels. Such type of hybrid microgels can be used as catalysts for various organic transformations because acrylamide moiety is the most inert one and does not interfere with organic species participating or producing in catalyzed organic reactions. The content and stability of metal nanoparticles within polymer network can be enhanced by their fabrication in P(NIPAM-Am-IM) microgels instead of P(NIPAM-Am) microgels. Therefore a lot of work on fabrication of metal nanoparticles within P(NIPAM-Am-IM) polymer microgels for various applications has been reported in modern literature.^9,14,17,26,65^

### 5.2. Use of P(NIPAM-Am) based microgels loaded with metal nanoparticles in surface enhanced Raman scattering

Noble metal nanoparticles stabilized by P(NIPAM-Am) microgels can be used in surface enhanced Raman scattering. Fang *et al.* used Fe_3_O_4_@P(NIPAM-Am)@Au hybrid microgels for this purpose.^8^ They recorded Raman spectra of 1 × 10^−6^ M crystal violet (CV) with and without of Fe_3_O_4_@P(NIPAM-Am)@Au hybrid microgels. A significant enhancement in intensity of the signals was observed in the presence of hybrid microgels. Thus noble metal nanoparticles fabricated P(NIPAM-Am) microgels are employed in surface enhanced Raman scattering.

### 5.3. Use of P(NIPAM-Am) based microgels in etalons

Smart polymer microgels have been widely used in photonics particularly in etalons.^15,68–71^ Etalons are modern interferometers. A dielectric medium is sandwiched between two reflecting surfaces in an etalon.^15^ P(NIPAM-Am) microgels acts as dielectric layer while metal nanoparticles film acts as reflecting surface in an etalon. If a light beam is incident upon an etalon, then it will be reflected at dielectric-reflecting interface many times as a result of which reflectance spectrum is obtained whose properties can be defined by the following equation

2 *mλ*_max_ = 2*nd* cos *θ*

where *m* is peak order, *λ*_max_ is wavelength associated to maximum reflectance, *d* is spacing between reflecting surfaces, *n* is refractive index of dielectric medium and *θ* is angle of incidence. This relation has been found valid for microgels based etalon. Microgels based etalons are more useful than that of other materials due to following reasons. Microgels possess responsive nature, so the spacing (thickness) and refractive index of the dielectric of etalon can be easily controlled by external stimuli like pH/temperature. On the other hand, it is difficult to change the thickness of etalon having non-responsive dielectric because if an undesired thickness of etalon has been built then it is of no use further. One has to rebuild the etalon for work. Microgels are elastic materials and contained large amount of water within their network. While other materials like graphite *etc.* are solid and possess high toughness. Therefore chances of scattering are very low in case of microgels based etalon as compared to that of other materials.

Sorrell *et al.* had proved the validity of this relation (eqn (1)) for a variety of etalons in which P(NIPAM-AA), P(NIPAM-Am) and P(NIPAM-VAA) microgels had been used as dielectric medium.^15^ They determined the effect of spacing (*d*), order (*m*) and refractive index (*n*) on the value of *λ*_max_. They also proved that value of *λ*_max_ was decreased with decrease in spacing. They increased the temperature of P(NIPAM-Am), P(NIPAM-AA) and P(NIPAM-VAA) microgels based etalon from 23 to 39 °C to decrease the size of microgel particle, so that the spacing of etalon would decreased resultantly. They also observed that value of *λ*_max_ and size of microgel particles both decreased sigmoidally with increase in temperature. It means a desired value of *λ*_max_ can be achieved by adjusting the value of *m*, *n*, *d* and *θ*. Sorrell *et al.* had prepared three microgel samples using different co-monomers.^15^ So their elasticity and refractive index are different from each other. But Sorrel *et al.* did not correlate the elasticity/refractive index of all the three microgels with *λ*_max_.^15^ Moreover they synthesized pH responsive microgel samples [P(NIPAM-AA) and P(NIPAM-VAA)] but they did not study the effect of pH on *λ*_max_. However pH stimulus could be used to modulate the spacing of etalon. Moreover they had carried out whole study at pH 4 at which pH responsive microgel samples are in deswollen state and carry no charge. The reflectance spectra should also be investigated under basic conditions (∼pH greater than p*K*_a_ of AA and VAA).

### 5.4. Use of P(NIPAM-Am) based polymer microgels in ordered surface structures

The use of microgel particle self-assemblies to generate periodic arrays (2D or 3D) onto some substrate like silicon wafer or metal film has gained a lot of attention due to their potential use in photonic, nano-fluidic and nano-lithography.^18,36^ To obtain periodic loosely packed (PLP) assemblies, a thin homogenous layer of microgel particles is coated on a substrate like silicon, metal or glass film. Then the microgel coating can be dried by free evaporation or droplet drying methods. The microgel coated surface is exposed to air at room temperature to dry the microgel layer in free evaporation method. While microgel coated surface is exposed to a hot flux of air for 10–15 seconds in droplet drying method to dry the microgel layer. Droplet drying method is more efficient as compared to free evaporation method. Free evaporation method took days while droplet drying method did same job within seconds. Moreover organized arrays of microgel particles on substrate are not formed by free evaporation method while completely organized arrays of microgel particles are formed by droplet drying method. That's why scientists prefer droplet drying method over conventional free evaporation method for this purpose. Angle of air flux and distance of air source from substrate can affect the arrangement of microgel particles on substrate. It has been studied that both of these parameters had little or no influence of microgel coating if drying is completed within 10–15 seconds. It has been also reported that closeness of microgel arrays only depends upon size of microgel particles. The arrangement of microgels particles is independent of their charge density, composition and sieve size. Horecha *et al.* has reported the aforementioned findings.^36^ They synthesized three silicon surfaces coated with P(NIPAM-Am), P(NIPAM-AA) and P(NIPAM-VAm) microgels. These samples possess different charge densities. But they studied that long range ordered arrays were formed by all samples which depicted that order of arrays did not depend upon charge. They also made porous coatings with the help of microgels. They sputtered microgel coated silicon wafer with nickel and gold films. Then they removed microgel layer by ultra-sonication. Thus pores were developed at place of microgel in nickel/gold films. They confirmed the formation of pores using SEM and AFM images.

### 5.5. Enzymes immobilization with tunable activity on modified P(NIPAM-Am) based microgels

**Fig. 4:**
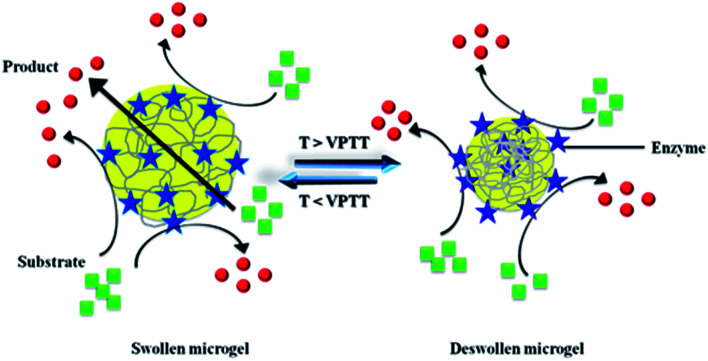
Catalytic conversion of substrate into product by enzyme molecules mobilized in responsive microgel at different temperatures.

Microgels possessing primary amine group (NH_2_) as pendant group are used as platform for immobilization of polymers like proteins and dyes. Recently three approaches have been used to synthesize primary amine terminated microgels: (1) post-polymerization Hoffmann rearrangement, (2) post-polymerization grafting of primary amine groups, and (2) co-polymerization of thermo-responsive monomers with cationic co-monomers. Series of microgels with same crosslinking, hydrodynamic diameter and other morphological parameters cannot be achieved through approach (2) and (3). Therefore approach I is considered better than other two approaches. P(NIPAM-Am) microgels possess amide group as terminal group. These amide groups can be successfully converted into amine groups through Hoffmann rearrangement. Shiroya *et al.* has synthesized aminated microgels by Hoffmann rearrangement and used them as platform for immobilization of trypsin enzyme.^72^ Initially they produced terminal amine groups on P(NIPAM-Am) microgels by Hoffman rearrangement. All the amide groups were not converted into amine groups by Hoffmann rearrangement. Then they oxidized the remaining amide groups into carboxyl groups by base hydrolysis. In this way they synthesized P(NIPAM-Am)-mod microgels which possess amine and carboxyl both as terminal groups. Then they immobilized the trypsin enzyme within microgels by carbodiimide coupling. Then they modulated the activity of enzyme by thermo-responsive swelling/deswelling of microgels. VPTT of P(NIPAM-Am) modified microgels was 33 °C while VPTT of trypsin-P(NIPAM-Am) modified microgels was ∼40 °C. They immobilized 2.2 and 4.1 mg trypsin in 1 g of microgels then they observed that enzymatic activity was decreased when temperature was increased above VPTT of microgels. Actually microgels got deswell with increase in temperature, so the diffusion of substrate molecules was decreased and enzymatic activity decreased resultantly. They also observed that enzymatic activity was decreased gradually when VPTT was approached while *R*_h_ of microgels was decreased sharply at that temperature. These results were indicated that some enzyme molecules present at peripheral region of microgel moved onto surface of microgel particles due to deswelling as shown in Fig. 4. So the enzymatic activity of microgels did not shut off completely. Enzyme molecules present at surface of microgels catalyzes the process. That's why the trypsin activity was decreased gradually around VPTT. Shiroya *et al.* has converted amide into amine group but it is clear from their results that all the amide groups had not been converted into amine groups by Hoffmann rearrangement.^72^ They did not study the pH responsive behavior of P(NIPAM-Am) modified microgels which might reveal the amphoteric nature of these microgels because amine and carboxyl both groups were present within same particle. They carried out complete study at pH 7, but they were not aware that either particle is in maximum swollen state here or not. Because VPT pH of amphoteric particles depends upon [NH_2_]/[COOH] ratio. As they did not report the number of amine and carboxyl groups in P(NIPAM-Am) modified microgels, so their study is not comprehensive.

Later Wang *et al.* reported all these parameters comprehensively.^35^ They discovered that all amide groups can be converted into amine groups completely if NIPAM was replaced by NIPMAM and Am was replaced by MAAm. They also reported that Hoffmann side reaction also occurred along with main reaction when P(NIPAM-Am) microgels were treated with bleach (reagent for Hoffmann reaction).^35^ Wang *et al.* also pointed out that crosslinker BIS hydrolyzed in the presence of bleach.^8^ Wang *et al.* had overcame these drawbacks by replacing P(NIPAM-Am) microgels with poly(*N*-isopropylmethacrylamide-methacrylamide) [P(NIPMAM-MAm)] as platform for enzymes immobilization.^35^ They also replaced crosslinker BIS with ethylene glycol dimethacrylate (EGDM). They prepared four different microgels: P(NIPAM-Am), P(NIPMAM-MAm), poly(*N*-isopropylmethacrylamide-methacrylamide-acrylic acid) [P(NIPMAM-MAm-AA)] and poly(*N*-isopropylmethacrylamide) [P(NIPMAM)]. P(NIPMAM-MAm), P(NIPMAM-MAm-AA) and P(NIPMAM) microgels were crosslinked by EGGM while P(NIPAM-Am) microgels were crosslinked by BIS. They treated all microgels with bleach in basic media. They found that crosslinker EDGM did not hydrolyze while crosslinker BIS hydrolyze in basic media. They treated P(NIPMAM-MAm) and P(NIPAM-Am) microgels with bleach and investigated their electrophoretic mobility. They found that P(NIPMAM-MAm) microgel sample did not give negative potential value at any pH value. While P(NIPAM-Am) microgels sample showed negative potential value after pH 8.5 (isoelectric point = 8.5 pH). This showed that Hoffman side reaction did not occur when P(NIPMAM-MAm) microgels was treated with bleach. They also investigated that 51% of amide groups were converted into amine groups during Hoffman rearrangement. They also studied the pH responsive behavior of samples after Hoffmann rearrangement. They measured the *R*_h_ values of particles using DLS instrument at different pH values of medium. They observed that *R*_h_ value of NIPMAM based sample was decreased with increase in pH of media. They also observed that *R*_h_ of NIPAM based sample was decreased with increase in pH up to pH = 8.5, when pH of medium was increased above 8.5 then *R*_h_ value of particles was started to increase. It meant, NIPMAM based sample showed basic behavior while NIPAM based sample showed amphoteric behavior. This result also confirmed that carboxyl groups were formed in NIPAM based sample only. They investigated that amount of amine groups formed depends upon time. Thus they prepared a series of amine terminated microgels by varying the time of reaction. They prepared samples by carrying the reaction for 10, 30 and 60 min. They found that 13%, 26% and 51% of the amide groups were converted into amine groups at 10, 30 and 60 min in P(NIPMAM-MAAm) sample respectively. They also treated P(NIPMAM-MAAm-AA) microgels with bleach for various time. Thus they prepared a series of samples having [NH_2_]/[COOH] ratio 0.4, 0.6 and 1.4 with isoelectric point at pH 4, 4.5 and 6.8 respectively. In this way they prepared series of amine terminated microgels of P(NIPMAM-MAm) and P(NIPMAM-MAm-AA) samples. Samples of these two series possess different number of amine groups only while morphological characters, *R*_h_, crosslinking density and monomers distribution in polymer were same. These attributes can be achieved in amine terminated microgels prepared through post-polymerization grafting and copolymerization with cationic co-monomers. They also treated all microgel samples with fluorescein isothiocyanate dye. They compared the UV-visible spectra of all samples and concluded that dye was attached to the microgel having amine group only. Because spectra of amine terminated microgel sample showed peak only while spectra of all other samples having no amine group showed no peak. Thus they practically studied the working of amine terminated microgels.

### 5.6. Use of P(NIPAM-Am) based microgels in catalysis

NiPAm and Am with some ionic co-monomer based hybrid microgels have also been extensively used as catalysts in different reactions due to high surface-to-volume ratio of inorganic nanoparticles loaded into the polymeric network.^4,13,20,26,27,42^ P(NIPAM-AAm) microgels may be an ideal carrier for nanoparticles to be used for catalysis due to inertness of functionalities of polymeric network. Pendant isopropyl and amide groups of NIPAM and Am units keep the nanoparticles fix inside the network without any aggregation. Crosslinking of network act as physical barrier against coalescence of nanoparticles. Moreover Am is hydrophilic so it increases the water holding capacity of network. So Am makes the catalyst more suitable for catalysis to be carried out in aqueous medium. Responsive microgels have ability to swell/deswell reversibly in response to various stimuli. The catalytic activity of nanoparticles fabricated within microgels can be controlled by swelling/deswelling of microgel network. Temperature is the reported trigger for swelling/deswelling of P(NIPAM-Am) hybrid microgels.^26,73^ Another important feature of P(NIPAM-Am) hybrid microgels is that the catalytic activity of the hybrid system can be tuned by varying temperature of the medium because NIPAM moiety of hybrid microgels becomes hydrophobic at high temperature as a result of which polymer network deswells. The shrinkage of the network creates a barrier in diffusion of reactant molecules towards the catalyst surface due to which rate of reaction decreases. Similarly polymer network swells at low temperature and allows the diffusion of reactants towards surface of nanoparticles to enhance the rate of reaction. On the other hand, amide pendant groups are present in close vicinity of nanoparticles in deswollen state, so they block the active sites of nanoparticles. Ajmal *et al.* have reported temperature and pH as triggers (stimuli) to control the catalytic activity of Ag–P(NIPAM-Am-MAA) hybrid microgels.^26^ They used NIPAM and MAA to make the microgels temperature and pH responsive at the same time. Catalytic reduction of 4-nitrophenol (4-NP) was carried out in basic medium. Carboxyl group of MAA is ionized at high pH (pH > p*K*_a_) and converted into negatively charged carboxylate ions which makes the carrier swollen and hydrophilic in basic medium. They investigated the catalytic reduction at 25 and 55 °C. They found 12 × 10^−2^ and 41 × 10^−2^ min^−1^ as value of apparent rate constant at 25 and 55 °C respectively. They also studied the temperature responsive behavior of microgels at various pH values. This study revealed that microgels are present in deswollen state at 55 °C and swollen state at 25 °C. So the value of rate constant at 55 °C should be smaller than that at 25 °C. But it was observed that the value of rate constant at 55 °C is greater than that of 25 °C. This is due to the Arrhenius behavior of reaction. They did not report the catalytic reduction of 4-NP at various temperatures, hence the effect of temperature on rate constant cannot be understood clearly from this study. Wu *et al.* have reported glucose as trigger for catalytic activity of Au nanoparticles.^73^ They synthesized glucose responsive Au–P(NIPAM-Am-VPBA) core–shell hybrid microgels. They studied effect of hydrophilic/hydrophobic state of microgel on the catalysis of 4-NP and nitrobenzene in aqueous medium. They studied that hybrid microgels are glucose responsive in 0–4 mM glucose concentration range only. They investigated that size of hybrid microgel increases with increase in glucose concentration up to 4 mM. While size of hybrid microgel was very slightly increased when glucose concentration was increased from 4 to 5 mM. They studied catalytic reduction of 4-NP and NB in 0–5 mM glucose concentration range. They concluded that value of apparent rate constant for reduction of 4-NP increases with increase in glucose concentration while value of *k*_app_ for reduction of NB decreases with increase in glucose concentration. Actually microgels are hydrophilic in swollen state, so they allow the diffusion of hydrophilic 4-NP and hinder the diffusion of hydrophobic NB and *vice versa*. That is why glucose concentration dependence of *k*_app_ of 4-NP reduction was found to be different from that of *k*_app_ of NB reduction. Thermally tunable catalytic reduction of 4-nitroanline using P(NIPAM-Am-MAA) hybrid microgels has been recently reported by our group.^24^

We are the pioneer who reported the use of Ag nanoparticles fabricated P(NIPAM-Am) hybrid microgels as catalyst for reduction of 4-nitrophenol in aqueous medium.^13^ Reduction of 4-NP was carried out at ambient temperature using sodium borohydride as reducing agent in presence of Ag–P(NIPAM-Am) hybrid microgels catalysts under various reaction conditions in aqueous medium. Our detailed studies on catalytic activity of NIPAM and Am based hybrid microgels can be found in literature.^13,26,27^ Catalytic activity of P(NIPAM-Am) based hybrid microgels has not been extensively explored in literature which stimulate us to carryout work on synthesis, characterization and catalytic applications of metal nanoparticles in P(NIPAM-Am) microgels for various organic reactions. Some work on this particular system has been already reported by us and more extensive study on it is in progress in our laboratory and will be published shortly.

### 5.7. Optical sensing applications of acrylamide based microgels

P(NIPAM-Am) microgels and P(NIPAM-Am-AA) microgels have a potential to be used as templates for immobilization of assemblies of azo dyes for optical sensing applications. Wu *et al.*^59^ used P(NIPAM-Am) microgels and P(NIPAM-Am-AA) microgels as support for loading of assembly of Calcon dye molecules. P(NIPAM-Am)-Calcon composite microgels P(NIPAM-Am-AA)-Calcon composite microgels were obtained by adding Calcon dye aqueous solution into pure microgels dispersion dropwise followed by two days stirring under N_2_ atmosphere and acidic pH conditions at room temperature. The composite microgels were purified by dialysis of the dispersion against distilled water. Absorption band of free Calcon solution is shifted towards longer wavelength with increase in pH but this shift is not systematic. Moreover a new band around 636 nm wavelength is found to appear in highly basic conditions. The absorption band of composite microgels is systematically shifted towards longer wavelength with increase in pH of medium and number of bands is same under acidic and basic pH conditions. The pH of medium is affected by concentration of H_2_O_2_. So the concentration of H_2_O_2_ can be easily sensed by the position of absorption band of composite microgels. In this way composite microgels play crucial role in optical sensing of H_2_O_2_.

Optical glucose sensing of NIPAM and Am based polymer microgels loaded with inorganic nanoparticles has been a subject of interest for a wide community of researchers.^74,75^ Biocompatible and fluorescent carbon dots (CDs) with average diameter of 6 nm loaded in glucose-imprinted poly(*N*-isopropylacrylamide-acrylamide-vinylphenylboronic acid) [P(NIPAM-AAm-VPBA)] polymer microgels can be used for optical detection of glucose under physiological conditions.^76^ The variation in glucose concentration can cause quenching in CDs signals in a reversible way and can be measured easily. The variation in intensity of fluorescent signal with variation of glucose concentration of surrounding medium is attributed to its complexation with PBA which results in swelling of polymeric network.

### 5.8. Biomedical applications of P(NIPAM-Am) based microgels

Smart polymeric material has gained a great deal of attention in biomedical applications and a flood of publications dealing with this area has been appeared in literature^77–88^ but our discussion is limited to biomedical applications of P(NIPAM-Am) and P(NIPAM-Am-IM) polymer microgels and their hybrids as given below.

#### 5.8.1. Thermotherapy or tumor hyperthermia

Thermotherapy is the controlled use of heat to treat cancer. Magnetic nanoparticles fabricated P(NIPAM-Am) microgels can be used to treat cancer cells. Fe_3_O_4_–P(NIPAM-Am)-Au hybrid microgels have been employed for this purpose.^8^ Temperature of the system increases with increase of exposure time of AC magnetic field over hybrid microgels as shown in Table 2. Temperature required in thermotherapy is 42–46 °C. Thus, Fe_3_O_4_–P(NIPAM-Am)-Au hybrid microgel can be used for destruction of tumor cells.

**Table 2:** Magneto caloric effect of Fe_3_O_4_–P(NIPAM-Am)-Au hybrid microgels

Time (s)	Temperature (°C)
65	27.5
150	32.0
225	35.0
300	37.5
375	40.0
450	42.5
525	43.5
600	44.2
675	44.7
750	45.0

**Fig. 5:**
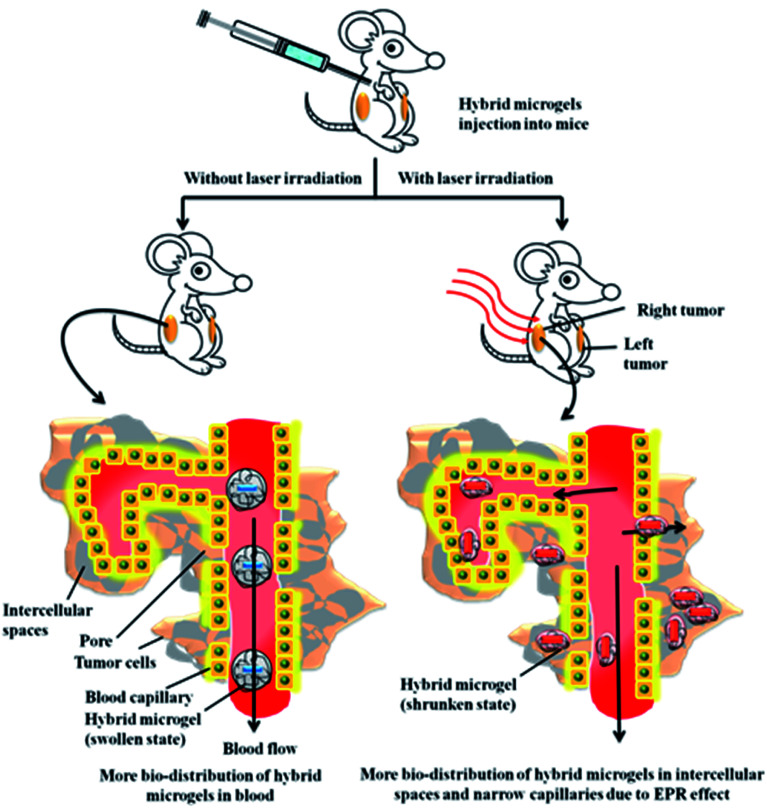
Distribution of hybrid microgels in mice in the presence/absence of laser irradiation.

There is another way through which P(NIPAM-Am) microgels can also be used for biomedical applications. P(NIPAM-Am) hybrid microgels can be loaded with Plasmonic nanoparticles which have tendency to absorb light radiations and convert this absorbed light energy into heat. Gold or silver nanoparticles possess SPR wavelength in visible range. It has been observed if radiation of SPR wavelength of nanoparticles is bombarded on hybrid microgel as a result microgels got deswell. This is termed as photo-thermal deswelling. Actually nanoparticles transform the light energy into heat energy and transfer this heat energy to microgels. So the temperature of microgels is increased and deswelling occurred. Shiotani *et al.* have studied the effect of photo-thermal deswelling on the bio-distribution of microgels within mice body^25^ (Fig. 5). They synthesized Au nanorods coated with poly(*N*-isopropylacrylamide-*N*,*N*-dimethylacrylamide) [P(NIPAM-DMAm)] and P(NIPAM-Am) microgels having VPTT around 37 and 45 °C respectively in aqueous medium. So Au–P(NIPAM-DMAm) microgels got deswell and Au–P(NIPAM-Am) did not deswell when injected into mice. That is why Au–P(NIPAM-Am) hybrid microgels are more suitable for photo-thermal treatment of tumors than Au–P(NIPAM-DMAm) hybrid microgels. They injected Au–P(NIPAM-Am) hybrid microgels into body and analyzed the % injection dose/gram of tissue in various body parts after 30 min and 72 h. They observed that maximum % injection dose/gram of tissue were present in blood and spleen after 30 min and in spleen after 72 hour of injection. They also irradiated the right tumor with near infra-red laser of intensity 3.8 W m^−2^ for 10 min but they did not irradiate the left tumor. They compared the % injection dose/gram of tissue of left and right tumor. They analyzed that hybrid microgels accumulated in right tumor was greater than that of left tumor. They explained it on the basis of enhanced permeability and retention (EPR) effect. As the right tumor was irradiated, so the hybrid microgels were in de-swollen and hydrophobic state in this tumor. So they got accumulated here in the fine capillaries, cellular spaces of vessels and extracellular matrix due to the hydrophobic interactions and very small size of hybrid microgels. They did not optimize various parameters like how much injection dose is sufficient, does surface structure of hybrid microgels suitable or not, *etc.* Their work was of basic nature. A lot of work is needed in this area. Scientists had reported the effect of photo-thermal treatment on tumor cells like Zhang *et al.* had studied the photo-thermal treatment of tumor cell in mice by Au–P(NIPAM-AA) hybrid microgels.^89^ But Au–P(NIPAM-AA) hybrid microgels are not neutral. They possess ionic co-monomer AA whose p*K*_a_ value is ∼4.5. The p*K*_a_ value of Ac is less than pH of blood ∼7.3. So Au–P(NIPAM-AA) microgels were converted into swollen and negatively charged state when injected into body. So Au–P(NIPAM-AA) microgels would not deswell easily on laser irradiation in comparison to Au–P(NIPAM-Am) microgels. Chemotherapy is an alternate methodology which is widely used for treatment of a variety of cancers. Cancer treatment efficiency can be significantly enhanced by combining both therapies in a single system. P(NIPAM-Am) based Chemo-photothermal therapy has been recently introduced.^90^ P(NIPAM-Am) polymer nanogel particles were fabricated within a liposome template to obtain core shell system with P(NIPAM-Am) thermosensitive core and liposome shell. An anticancer drug doxorubicin hydrochloride and a photosensitizer (indocyanine green) both were encapsulated within P(NIPAM-Am) core by ammonium sulfate gradient methodology. Upon irradiation, Indocyanine green converts light energy into heat energy to create hyperthermia and to induce deswelling in polymer core simultaneously which results in quick release of anticancer drug to tumor cells. The presence of liposome layer around P(NIPAM-Am) nanogel particle may cause barrier in release of anticancer drug in above described system. However rate of release of drug from P(NIPAM-Am) can be increased by changing the morphology of P(NIPAM-Am) based anticancer drug delivery system. For example SiO_2_ core containing ZnS : Mn^2+^ and Fe_2_O_3_ quantum dots can be covered by P(NIPAM-Am) shell to design more facile anticancer drug release system as reported by Cao *et al.*^91^

Cytotoxicity of microgels must be studied before *in vivo* application of microgels. Wu *et al.*^14^ has reported the cytotoxicity of P(NIPAM-Am) microgels. They synthesized Ag nanoparticles of diameter 40 nm loaded them into poly(*N*-isopropylacrylamide-acrylamide-4-vinyl phenyl boronic acid) [P(NIPAM-Am-VPBA)] microgels. They studied the viability of B16F10 liver cells in the presence of hybrid microgels and naked Ag nanoparticles. It is well known fact that silver nanoparticles possess cytotoxic nature. They observed that cells were remained viable 100% in the presence of hybrid microgels but the viability of cells was decreased in the presence of naked nanoparticles. So it was concluded that cytotoxic nature of silver nanoparticles has been masked by the microgel network. Their results very well support that hybrid microgels can be successfully used for the *in vivo* drug delivery and bio-sensing.

#### 5.8.2. Stimuli responsive drug release from acrylamide based microgels

Acrylamide based temperature responsive colloidal microgels are frequently used for drug delivery.^16^ The microgels loaded with drug molecules can be injected easily to target cells in the form of aqueous suspension as compared to other macroscopic hydrogels. Moreover microgels have quick response as compared to macro gels due to which they have been widely used as drug delivery systems. Different temperature and pH responsive microgels based on acrylamide moieties have been reported for controlled drug delivery to target cells.^11,92^

Sung *et al.* reported crosslinked gelatin based microgels loaded with P(NIPAM-Am) polymer chain as biocompatible and temperature controlled drug delivery system of bovine serum albumin (BSaN).^11^ They investigated the kinetics of release of BSaN from aforementioned microgel system under heating and cooling cycles at 22 and 42 °C and reported that more than 60% drug can be released from carrier after four heating and cooling cycles within 3 hours while only 10% release is obtained at constant temperature of 22 °C in the same time. The increase in drug release with increase in temperature may be attributed to conversion of coil to globule conformation of P(NIPAM-Am) minor component of microgel system. This change in conformation causes reduction in volume of elastic gelatin microgel particles which enhances the convective flow of drug from polymer network to outside. They also reported that biocompatibility and rate of drug release from microgels can be tuned by concentration of P(NIPAM-Am) and extent of crosslinking of gelatin matrix. They claimed that this drug delivery system is biodegradable but in fact P(NIPAM-Am) component is not biodegradable. However they used this component in very small quantity which can be excreted by kidney and can be removed *via* renal filtration.

Wang *et al.*^12^ used P(NIPAM-Am) microgels as carrier of water soluble bleomycin which is an anti-cancer drug used for chemotherapy. The microgels were loaded with drug to obtain uniform suspension. Progress of drug release from microgel suspension was monitored by UV-visible spectrophotometry by measuring absorbance at 295 nm. It was concluded that rate of drug release is strongly dependent upon composition and concentration of P(NIPAM-Am) microgel particles. Moreover drug release from microgel system follows Peppas empirical equation and occurs according to Fickian diffusion. They reported that 100% release of drug from microgel system can be obtained within 25 hours. A rapid delivery of Indomethacin drug from P(NIPAM-Am-HEA) microgel particles with 100% release within just 100 minutes has been reported by Fundueanu *et al.*^16^ but this study was carried out in methanol instead of aqueous medium which limits its biomedical practical applications. A lot of many other publications on drug delivery systems based on Nipam and Am are available in scientific literature^75,92,93^ but acrylamide unit of such kind of microgels is toxic and its toxicity has not been properly addressed in this literature. The low level biodegradability and biocompatibility makes these microgels less desired systems for targeted drug delivery because its long term storage in body causes cytotoxicity. More over in aqueous medium, P(NIPAM-Am) microgels aggregate at temperature > LCST and limits their use as drug carrier. However P(NIPAM-Am-IM) microgels are more hydrophilic and may be a better candidate of drug delivery system due to their high stability in a wide temperature range. Therefore P(NIPAM-Am-IM) microgels have been widely reported as drug delivery systems.

## 6. Summary and future perspective

Poly(*N*-isopropylacrylamide-acrylamide) microgels are important microgels due to their certain characteristics like responsive nature, hydrophilic and inert character as well as their specific volume phase transition temperature that lies around 37 °C. Moreover, in P(NIPAM-Am) microgels, individual properties of NIPAM and Am are maintained. These properties enlarged the usage of these specific microgels in different fields. P(NIPAM-Am) microgels have been used in various applications like polymer coating and immobilization, use as micro-reactor for stabilization of nanoparticles, catalysis, sensing of glucose, drug delivery and etalons. The amide group of P(NIPAM-Am) microgels can be converted into amine group successfully and amine terminated microgels can successfully bind polymers (enzymes) and dyes. These microgels are non-cytotoxic at their low concentrations and possess volume phase transition temperature around 37 °C. So they have been used in drug delivery, bio-sensing and photo-thermal treatment. P(NIPAM-Am) microgels can develop into a long-range ordered thin layer at metal film, so they have been used in coating. Microgel layer is sandwiched between two metal film layers to made optical device. As the dielectric of the microgel (hydrophobic/hydrophilic) and distance between two metal plates is controlled by size of microgel, so these microgels are largely used in etalons. So metal nanoparticles fabricated P(NIPAM-Am) hybrid microgel can be used in tumor hyperthermia^8^ and spectroscopic analysis. We believe that acrylamide based microgels will gain more and more attention in future due to their special feature of functional colloids. However few aspects of P(NIPAM-Am) and P(NIPAM-Am-IM) polymer microgels have not been properly explored. For example biocompatibility and biodegradability of P(NIPAM-Am) and P(NIPAM-Am-IM) polymer microgels should be studied in detail to practically apply them in biomedical field. Various polymer based materials have been reported for environmental applications to extract/degrade toxic substances^94–104^ but this aspect of P(NIPAM-Am) and P(NIPAM-Am-IM) microgels has been rarely explored in literature. Silver nanoparticles loaded polymeric material has been extensively reported for antibacterial applications^105–107^ but antibacterial behavior of silver nanoparticles loaded in P(NIPAM-Am) and P(NIPAM-Am-IM) microgels may be a subject of future studies in this area.

## Conflicts of interest

There are no conflicts to declare.

## List of abbreviations

IM: Ionic moiety
VA: Vinylamine
LCST: Lower critical solution temperature
NIPAM: *N*-Isopropylacrylamide
NIPMAM: *N*-Isopropylmethacrylamide
Am: Acrylamide
VAA: Vinyl acetic acid
AA: Acrylic acid
MAA: Methacrylic acid
HEA: Hydroxyethyl acylate
IPN: Interpenetrating network
PBA: Phenylboronic acid
VPBA: 4-Vinyl phenyl boronic acid
VPTT: Volume phase transition temperature
DMAm: *N*,*N*-Dimethylacrylamide
KPS: Potassium per sulfate
APS: Ammonium per sulfate
AiBN: Azodiisobutyronitrile
MBAAm: Methylene bis acrylamide
TEMED: *N*,*N*,*N*′,*N*′-Tetramethylethylene-diamine
EGDM: Ethylene glycol dimethacrylate
NaPS: Sodium per sulfate
SDS: Sodium dodecyl sulfate
TEM: Transmission electron microscopy
DLS: Dynamic light scattering
UV-vis: UV-visible spectroscopy
FTIR: Fourier transform Infrared spectroscopy
SEM: Scanning electron microscopy
AFM: Atomic force microscopy
EDX: Energy dispersive X-ray spectroscopy
RS: Raman spectroscopy
NMR: Nuclear magnetic resonance
DMA: Differential mechanical analysis
LLS: Laser light scattering
DSC: Differential scanning calorimetry
TGA: Thermogravimetric analysis
GT: Gelling temperature
CPT: Cloud point temperature
VPBA: Vinyl phenyl boronic acid
BSaN: Bovine serum albumin

## Supplementary Material
